# Supplementary material for: Efficient Regeneration of Transgenic Rice from Embryogenic Callus via Agrobacterium-Mediated Transformation: A Case Study Using GFP and Apple MdFT1 Genes
Source: Plants (Basel). 2024 Oct 6;13(19):2803. doi: 10.3390/plants13192803 (PMC11478628; doi:10.3390/plants13192803)
Supplement: Supplementary file 1 [file plants-13-02803-s001.zip › Supplementary Materials_Figure S1-S5.pdf]

plants

Suppementary Materials

MDPI

Article

Efficient Regeneration of Transgenic Rice from Embryogenic Callus via *Agrobacterium*-Mediated Transformation: A Case Study using *GFP* and Apple *MdFT1* Genes

Van Giap Do[1,*], Seonae Kim[1], Nay Myo Win[1], Soon-Il Kwon[1], Hunjoong Kweon[1], Sangjin Yang[1], Juhyeon Park[1], Gyungran Do[2] and Youngsuk Lee[1,*]

[1] Apple Research Center, National Institute of Horticultural and Herbal Science, Rural Development Administration, Daegu 43100, Republic of Korea; seonaekim@korea.kr (S.K); naymyowin@korea.kr (N.M.W.); topapple@korea.kr (S.-I.K.); kweonhj@korea.kr (H.K.); yangsangjin@korea.kr (S.Y.); wngus1113@korea.kr (J.P.)

[2] Postharvest Research Division, National Institute of Horticultural and Herbal Science, Rural Development Administration, Wanju-gun 55365, Republic of Korea; microdo@korea.kr (G.D.)

[*] Correspondence: giapbio@korea.kr (V.G.D.); kongfo@korea.kr (Y.L.)

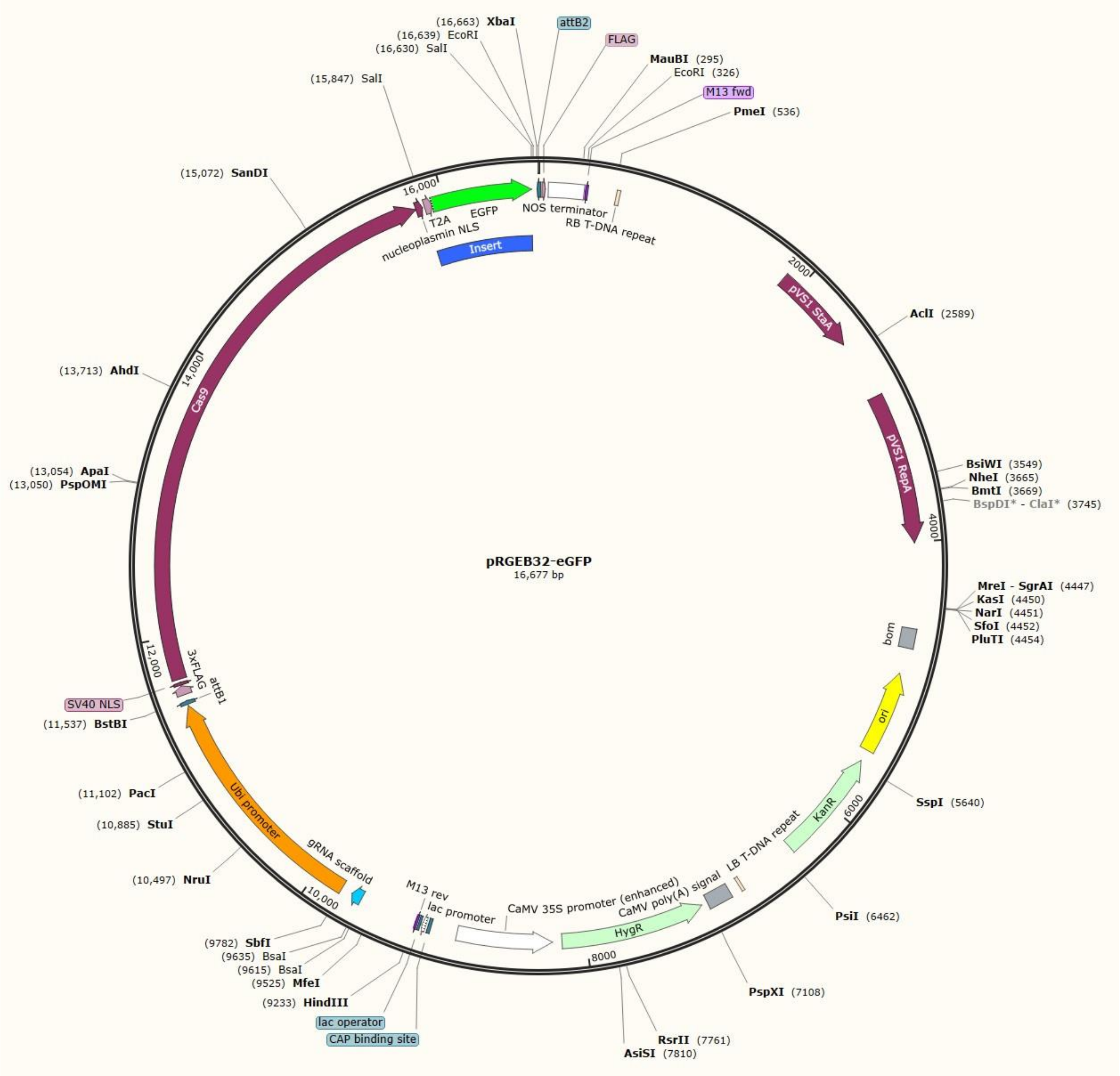

Figure S1. Full map of the recombinant expression vector Ubi::Cas9–eGFP.

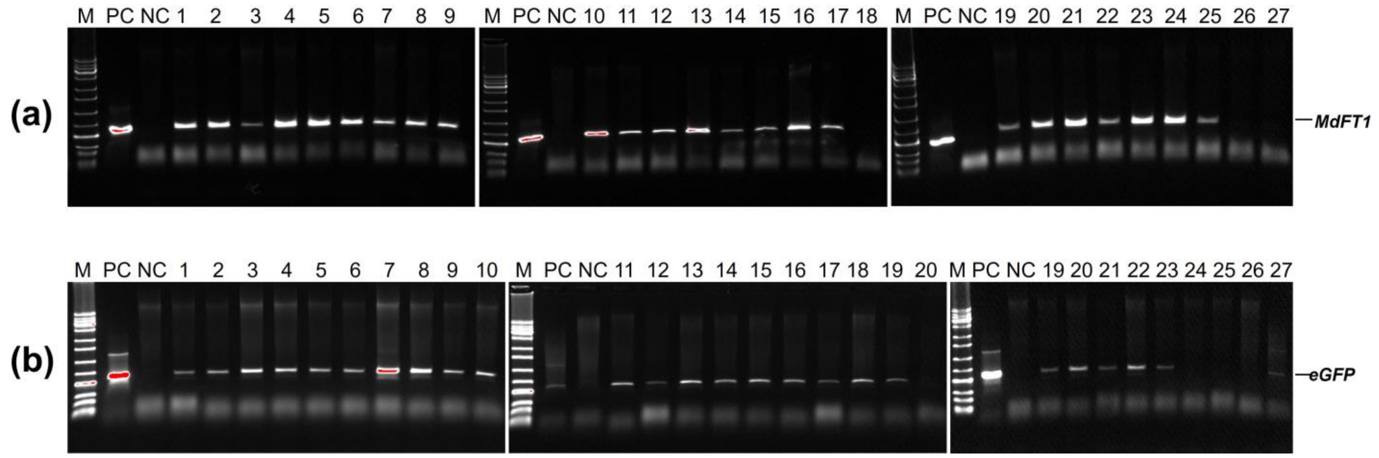

Figure S2. PCR-based screening of transgenic rice callus lines expressing Ram3D::MdFT1 (**a**) and Ubi::Cas9–eGFP (**b**). For screening of transgenic rice callus lines, 27 putative transgenic rice callus lines of each expression vector (which grown on the selection medium N6SE), were selected for genomic DNA PCR. **M**, 1 Kb plus DNA ladder; **PC**, plasmid DNA of 3D::MdTFL1 and Ubi::Cas9–eGFP (isolated from *E. coli*) as positive control; **NC**, genomic DNA of WT callus lines as negative control.

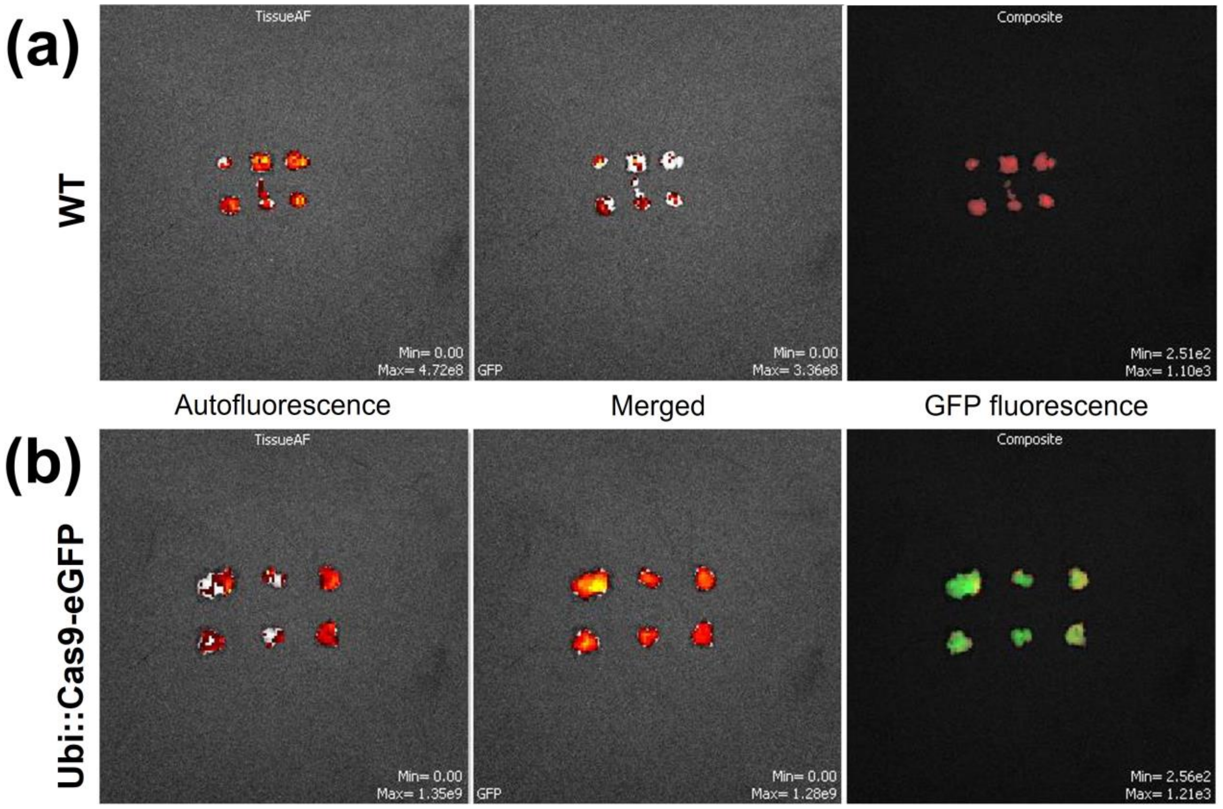

Figure S3. Observation of green fluorescent protein (eGFP) in rice calli of WT (**a**) and transgenic Ubi::Cas9–eGFP lines (**b**).

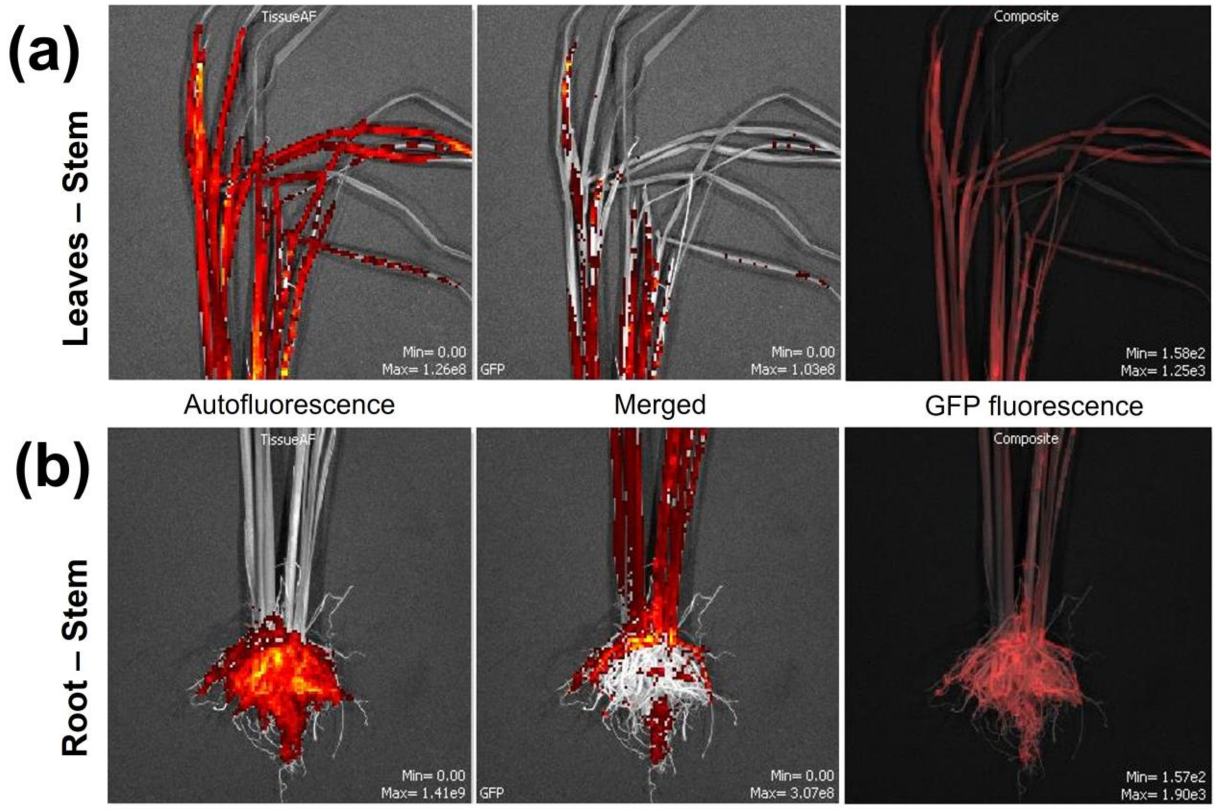

Figure S4. Observation of green fluorescent protein (eGFP) in different organs, including leaves–stem (**a**) and root–stems (**b**) in WT rice plants (T1 seedlings).

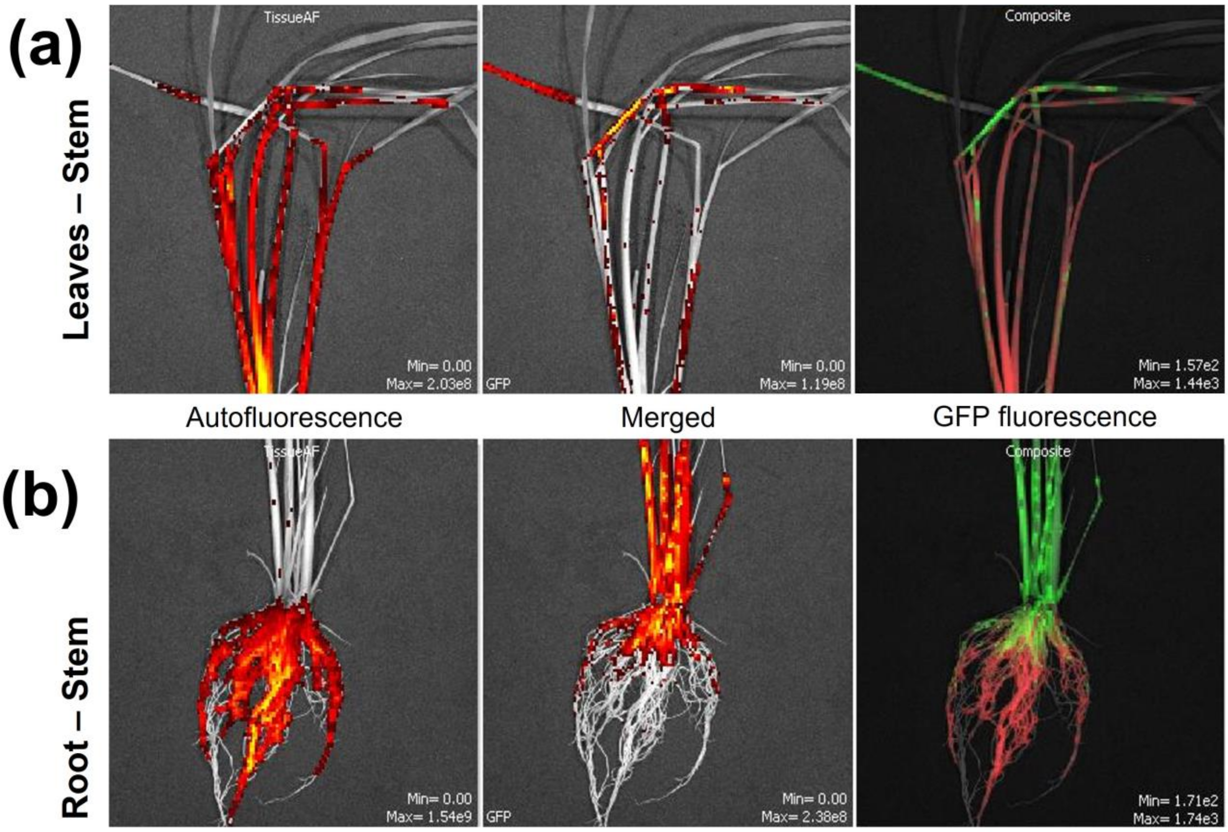

Figure S5. Observation of green fluorescent protein (eGFP) in different organs, including leaves–stem (**a**) and root–stems (**b**) in transgenic Ubi::Cas9–eGFP rice plants (T1 seedlings).
